# Supplementary figures and images for: Illuminating protist diversity in pitcher plants and bromeliad tanks
Source: PLoS One. 2022 Jul 27;17(7):e0270913. doi: 10.1371/journal.pone.0270913 (PMC9328516; doi:10.1371/journal.pone.0270913)

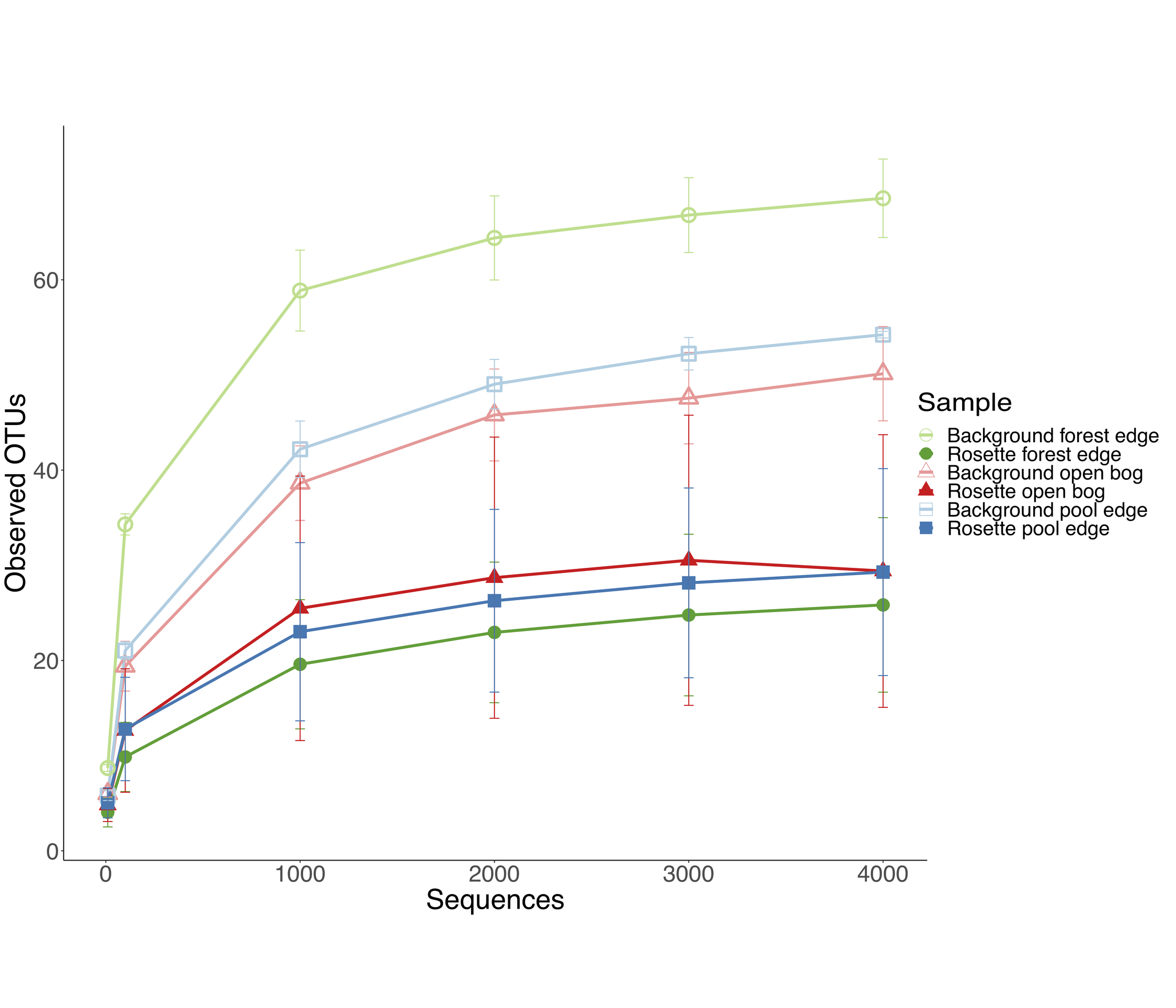

Supplement: S1 Fig — Communities from background water samples (open symbols) are consistently more species rich than from Sarracenia pitchers at Hawley Bog. The background water at the forest edge (light green) is consistently more species rich than the mid bog sites (pink and light blue) along our transect. Error bars are standard deviations. (TIF) [file pone.0270913.s001.tif]

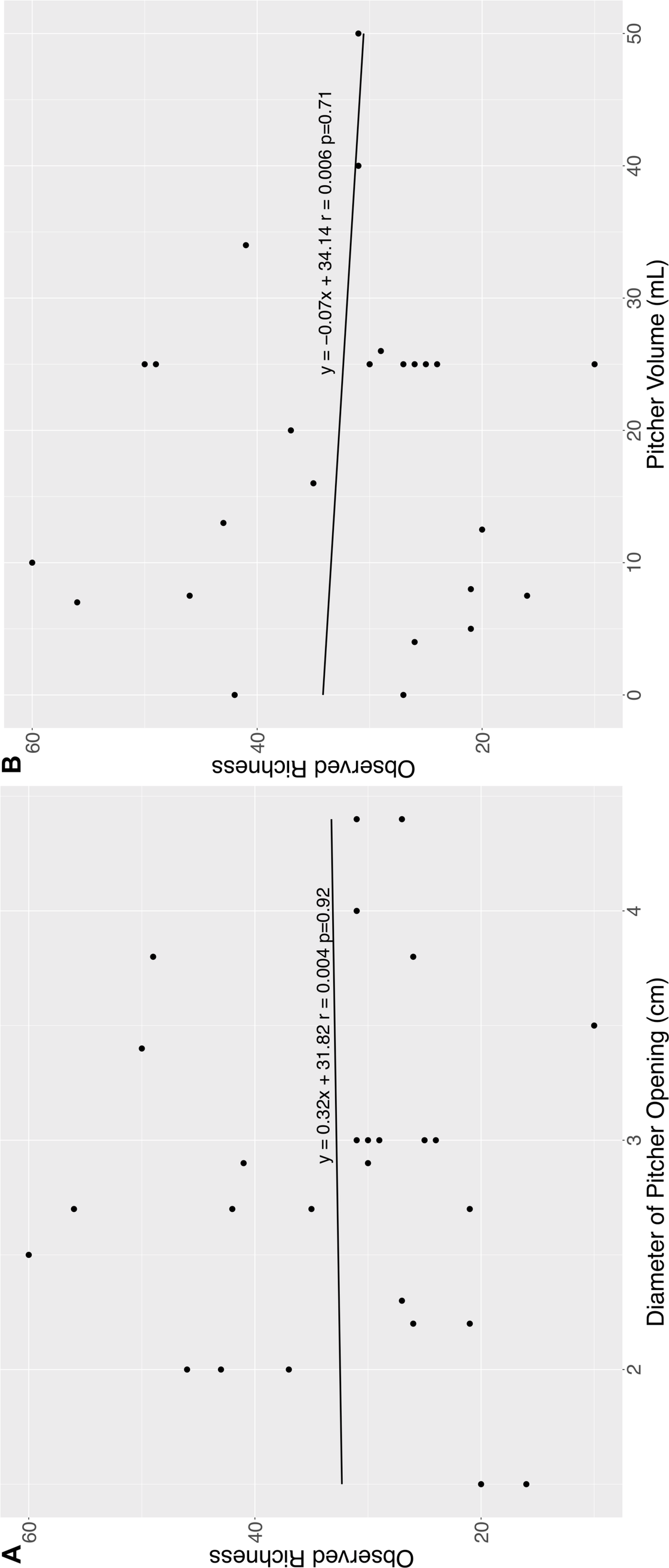

Supplement: S2 Fig — (TIF) [file pone.0270913.s002.tif]

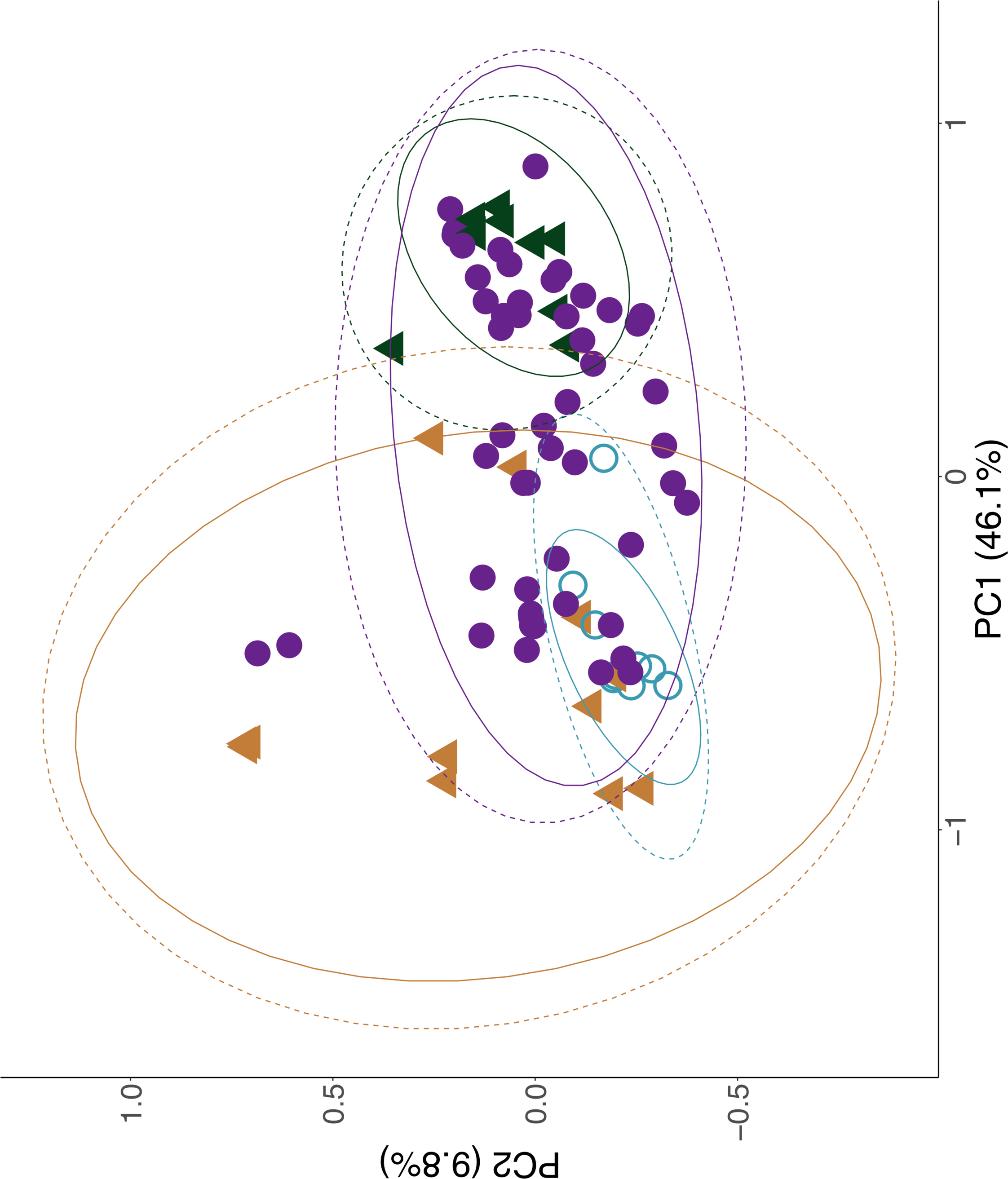

Supplement: S3 Fig — (TIF) [file pone.0270913.s003.tif]
