# Supplementary material for: Illuminating protist diversity in pitcher plants and bromeliad tanks
Source: PLoS One. 2022 Jul 27;17(7):e0270913. doi: 10.1371/journal.pone.0270913 (PMC9328516; doi:10.1371/journal.pone.0270913)
Supplement: Attachment — Submitted filename: Response to Reviewers_LAK_RSS.docx [file pone.0270913.s007.docx]

We thank the reviewers for their helpful comments, which we have addressed in detail below.

****

**Reviewer #3:**

Thank you very much for the revisions to the manuscript. I commend the authors for the work they did. Overall, I find the study interesting and timely. Eukaryotic diversity is understudied in general, and especially in pitcher plant systems. I still, however, have concerns about the methods in Study 2. My concerns do not mean that there is a problem with the data, but rather in the question that is being addressed (Succession). My reasons for this are detailed below:

As a result of the comments of this reviewer, we have further clarified the language in our manuscript including reframing successional patterns as seasonal change to the community over time.

1) Standardization of pitcher leaf age:

Sampling in the beginning of May in Massachusetts seems very early in the growing season for Sarracenia purpurea at this latitude. It is thus surprising that there were new leaves/pitchers available on the rosettes (especially 3 new leaves per rosette). More surprising is that the new leaves that were used in the study were open and filled with large volumes of water (as shown in the supplemental data), unless several large rainstorms occurred just before sampling. I just can’t imagine that the new pitchers (from that season) would be large enough to hold such a large volume at this time period.

Not all pitchers die back each year. Instead, they overwinter, with the snow protecting them. There will, of course, be some old pitchers from several seasons in the past that have died back (the pitchers closer to the ground), but the middle of the rosette will have pitchers from last season that have survived the winter as well as the new pitchers that are growing in the current season. It can sometimes be difficult to determine last season’s pitchers from new growth pitchers if the new growth pitchers are ~4 weeks or older. I am therefore uncertain if the authors used pitchers from that season or overwintered pitchers. For the study to examine Succession, the age and opening time of the pitchers needed to be better standardized. This can be done by selecting very new (fresh, flexible green leaves that had just opened) pitchers. The first sampling event should have then been done several weeks after they opened, so that there was time for the pitchers to fill with water.

REPLY: We thank the reviewer for these comments. We have reviewed field photos and believe it is possible that pitchers from the previous season were sampled, given how early in the season it was, and the size of some of the pitchers. We now clarify that the pitchers we sample may be of different generations, and that seasonality refers to turn over of the microbial community from spring through summer. We have also added that variance among spring pitchers might be due to the age of individual pitchers (i.e. those that overwintered vs new growth).

Study 2 still informs us about the inquiline community that inhabits pitchers, but without the initial standardization, it acts more of a snapshot at each point in the season, and not succession.

REPLY: We believe that our improved language has clarified this issue.

Apologies if my phenology is incorrect. Addition of field site information (flowering time, new leaf production, precipitation) should be added to the manuscript.

REPLY: We agree that to understand how the age and phenology of the pitcher plant impacts the inquiline community (e.g. Armitage 2017) requires repeated sampling of pitchers with standardized ages. However, our current study is interested in how the microbial community changes during the spring growing season, irrespective of pitcher age (e.g. Buosi et al. 2015). Pitcher age may be a factor in structuring these communities, but our current study demonstrates that seasonality tends to be a more important driver, given that communities in leaves of different ages converge by the end of the sampling window (Figure 4), as opposed to communities being structured by pitcher age. Nevertheless, to avoid giving a false impression we have removed the term succession when discussing our results and simply use the term seasonal changes. We have also clarified the age of pitchers and sampling strategy as well as site information in the Methods and Discussion.

2) Dipteran larvae in the inquiline community:

The large volume of water that was removed from each pitcher during sampling would also remove any of the dipteran larvae that are essential for dynamics of the inquiline community. Some (the midge and some of the mosquito larvae) could be down at the bottom of the leaf near the decomposing insects, so it is likely that those ones could remain in the community after each sampling. It is unfortunate that the authors did not record midge and mosquito larvae during the sampling so that the authors have an idea of how many were lost from the community during each sampling. Were there any mosquito larvae stuck on the filter after the inquiline community was filtered?

Along those lines, a better reference than Rango 1999 for seasonal Wyeomyia dynamics would be to use the Harvard Forest Data Archives. These archives contain data from the authors’ field site and/or sites in the region and have information about the seasonal dynamics of the inquiline mosquito larvae in the system. https://harvardforest1.fas.harvard.edu/exist/apps/datasets/showData.html?id=hf193

It very well might be that the mosquito larvae densities do not peak until mid-summer, and therefore are not a part of the May-June sampling that occurred in this study. In the lower latitude part of Sarracenia purpurea’s distribution (e.g., Florida) the mosquito larvae are the most abundant as soon as the leaf opens. If that is the case in this study site as well, then the mosquito density would be greatly impacted. If overwintered leaves were indeed used for this study, there could be mosquito larvae present because they were in diapause in the leaves during the winter.

REPLY: We appreciate this insight and have looked at the Ellison & Gotelli dataset. The hf193-04: food web structure data show that for Hawley Bog (as well as Molly Bog) the density for *Wyeomyia* peaks in August and September (with some detections in July, and very few in June). We have added this information to the discussion.

3) Disturbance by removing large volumes of water during sampling: a large volume was taken from each community during each sampling time period in Study 2. The authors do replace the volume they removed with the supernatant, but this is still a large, open niche space. I agree that there will be some microbes that remain at the bottom of the leaf during each sampling time period, but the community is undergoing a great disturbance during each sampling period, with diversity potentially being diluted each time.

In summary, I do think this study has accomplished a lot of things and is interesting, it is just that there are some potential issues in the methods which will affect the focus of the paper as it currently is written. In general, I suggest that the authors be careful with the word “succession” in this study. Instead, I suggest that the authors use “seasonal” dynamics or something along those lines.

REPLY: The volume of fluid removed does represent a disturbance to the system, and we have added additional text addressing this issue in lines 388-398 of the Discussion. The final sampling window shows that communities in the pitcher plants on Day 53 were quite similar to the background communities (Figure 4, yellow filled symbols are clustered with open fill bog water samples). If we were diluting diversity and changing the community composition drastically, we would expect the communities to be quite unique and would expect samples from Day 53 to cluster on their own near no natural samples. We contend that the dynamic/stochastic nature of the pitcher system is such that the disturbance created by our sampling is not beyond the scope of what is possible in nature, and therefore our results are a useful addition in the study of microbial eukaryote communities. We hope that this study inspires others to track additional communities using other experimental methods.
